# Prevalence, Awareness, Treatment, and Control of High LDL Cholesterol in New York City, 2004

**Published:** 2010-04-15

**Authors:** Ushma D. Upadhyay, Elizabeth Needham Waddell, Stephanie Young, Bonnie D. Kerker, Magdalena Berger, Thomas Matte, Sonia Y. Angell

**Affiliations:** New York City Department of Health and Mental Hygiene, New York, New York. Dr Upadhyay is also affiliated with the University of California San Francisco School of Medicine, San Francisco, California; Bureau of Epidemiology Services, New York City Department of Health and Mental Hygiene; New York City Department of Health and Mental Hygiene, New York, New York; New York City Department of Health and Mental Hygiene, New York, New York; New York City Department of Health and Mental Hygiene, New York, New York; New York City Department of Health and Mental Hygiene, New York, New York; New York City Department of Health and Mental Hygiene, New York, New York

## Abstract

**Introduction:**

Low-density lipoprotein (LDL) cholesterol is a major contributor to coronary heart disease and the primary target of cholesterol-lowering therapy. Substantial disparities in cholesterol control exist nationally, but it is unclear how these patterns vary locally.

**Methods:**

We estimated the prevalence, awareness, treatment, and control of high LDL cholesterol using data from a unique local survey of New York City's diverse population. The New York City Health and Nutrition Examination Survey 2004 was administered to a probability sample of New York City adults. The National Health and Nutrition Examination Survey 2003-2004 was used for comparison. High LDL cholesterol and coronary heart disease risk were defined using National Cholesterol Education Program Adult Treatment Panel III (ATP III) guidelines.

**Results:**

Mean LDL cholesterol levels in New York City and nationally were similar. In New York City, 28% of adults had high LDL cholesterol, 71% of whom were aware of their condition. Most aware adults reported modifying their diet or activity level (88%), 64% took medication, and 44% had their condition under control. More aware adults in the low ATP III risk group than those in higher risk groups had controlled LDL cholesterol (71% vs 33%-42%); more whites than blacks and Hispanics had controlled LDL cholesterol (53% vs 31% and 32%, respectively).

**Conclusions:**

High prevalence of high LDL cholesterol and inadequate treatment and control contribute to preventable illness and death, especially among those at highest risk. Population approaches — such as making the food environment more heart-healthy — and aggressive clinical management of cholesterol levels are needed.

## Introduction

Coronary heart disease (CHD) is the leading cause of death in the United States, accounting for 27% of all deaths in 2005 (1). An established body of evidence points to reducing low-density lipoprotein (LDL) cholesterol as one of the most effective ways to prevent and treat CHD, regardless of a person's risk (2-4). On average, every 1% reduction in LDL cholesterol is matched by a 1% reduction in the likelihood of a major cardiac event (5). Thus, small reductions in population LDL cholesterol could prevent many CHD-related deaths.

Despite advances in lowering total blood cholesterol, particularly throughout the 1980s (6,7), and the recent broad-scale use of medications targeting LDL cholesterol, control of lipid levels remains poor in the United States. Prevalence of high total cholesterol and high LDL cholesterol remained virtually unchanged between 1988-1994 and 1999-2004 (8,9), and only one-fourth of US adults with elevated LDL cholesterol have their condition appropriately controlled (8). Blacks and Mexican Americans are less likely than whites to take drugs from the statin class, and they have poorer control (8,10). National estimates of high LDL cholesterol are not available for other Hispanics or for Asians.

Local monitoring of the prevalence, treatment, and control of CHD risk factors is needed for planning and evaluating interventions to prevent disease. Previous studies suggest that New York City is similar to or better than the rest of the country in terms of prevalence and management of some CHD risk factors (hypertension and obesity) (11,12) but worse for others (diabetes) (12,13). However, no study has examined LDL cholesterol levels by using a representative sample in New York City or in any exclusively urban setting. In this study, we examine prevalence, awareness, treatment, and control of high LDL cholesterol in New York City adults by using the first community Health and Nutrition Examination Survey (NYC HANES). To define high LDL levels, we used the National Cholesterol Education Program Adult Treatment Panel III (ATP III) guidelines, which provide thresholds for diagnosing and targets for lowering high LDL cholesterol on the basis of individual CHD risk (5,14). Findings on variation in LDL cholesterol levels in this population may be useful for researchers and policy makers in other urban environments.

## Methods

NYC HANES is a population-based, cross-sectional, examination survey of noninstitutionalized New York City adult residents aged 20 years or older. A 3-stage cluster sampling design was used to recruit participants from June through December 2004. Detailed study methods are published elsewhere (15). The survey consisted of personal interviews, physical examinations, and laboratory testing. All survey instruments, protocols, and measurements were standardized to National Health and Nutrition Examination Survey (NHANES) specifications. Lipid profiles were analyzed at the Lipoprotein Analytical Laboratory at Johns Hopkins University Hospital. Most laboratories that perform testing for NHANES were used for NYC HANES (16).

The NYC HANES protocol was approved by the New York City Department of Health and Mental Hygiene and the New York State Department of Health institutional review boards. Study participants provided written, informed consent.

A total of 3,047 eligible participants were identified (84% household contact rate); 1,999 completed the face-to-face interview and at least 1 examination measurement (66% participant response rate), yielding an overall response rate of 55%. Of the 1,999 participants, valid high-density lipoprotein (HDL) and total cholesterol measurements were obtained for 1,783 participants. A random sample of participants (80%) was assigned to fast for at least 8 hours before giving blood, and of these, valid measurements were available for 1,150 participants. An additional 136 participants not assigned to fast but who did voluntarily were similar to those assigned to fast for all demographic characteristics except age and were included in the sample. Valid LDL cholesterol data were available for 1,286 participants.

### ATP III risk groups

Participants were categorized into 1 of 4 CHD risk groups — high, moderately high, moderate, or low — following ATP III guidelines (14). These consider 1) presence of CHD or CHD risk equivalents, 2) presence of CHD risk factors, and 3) 10-year CHD risk using Framingham risk scoring (Table 1).

CHD and CHD risk equivalents were self-reported history of congestive heart failure, coronary heart disease, angina pectoris, myocardial infarction, stroke, or diabetes (determined by self-report or having a fasting glucose value ≥126 mg/dL). CHD risk factors were self-reported cigarette smoking, hypertension (measured blood pressure ≥140/90 mm Hg or self-reported current use of antihypertensive medications) (11), measured HDL cholesterol <40 mg/dL, family history of premature CHD (reported knowledge of heart attack or angina before age 50 among biological grandparents, parents, or siblings), and older age (men ≥45 y, women ≥55 y). Measured HDL cholesterol ≥60 mg/dL was considered protective and offset the presence of 1 risk factor. The Framingham risk score estimates risk of developing angina pectoris, myocardial infarction, or CHD death within 10 years (17) and was calculated for each participant by using algorithms provided in ATP III guidelines.

### Cholesterol levels

Serum total and HDL cholesterol concentration were measured directly (12). LDL cholesterol was calculated from the fasting subsample's sera by using the Friedewald equation (18):

LDL cholesterol [mg/dL] = total cholesterol [mg/dL] – HDL [mg/dL] – triglycerides [mg/dL]/5.

High LDL cholesterol was defined as currently taking cholesterol-lowering medications or having LDL cholesterol levels at or greater than the ATP III risk-specific thresholds recommended for initiation of drug therapy in each risk group (5) (Table 1).

### Awareness, treatment, and control of high LDL cholesterol

Participants were asked if they had ever had their cholesterol checked, and if so, whether a doctor or other health professional had told them their cholesterol level was high. Those who answered yes were considered aware of their diagnosis of high cholesterol and then asked the following questions about treatment: "To lower your blood cholesterol, have you ever been told by a doctor or other health professional to: a) eat fewer high-fat or high-cholesterol foods, b) control your weight or lose weight, c) increase your physical activity or exercise, or d) take prescribed medicine?" Respondents who answered yes to any of the questions were asked whether they were following this advice. Those who reported following at least 1 of these recommendations were classified as having adopted a healthier behavior to lower their cholesterol. Those who said they were taking prescribed cholesterol-lowering medications were classified as treated with medication. For all participants with high LDL cholesterol, control was defined as measured LDL cholesterol lower than the ATP III goal for their specific risk group (Table 1).

### Demographics and health characteristics

NYC HANES assessed basic demographic characteristics (age, sex, race/ethnicity, place of birth, income, and education), health insurance status, cigarette smoking, and physical activity. We used the NHANES definition of "US-born," which includes the 50 states and Washington, DC; participants born in Puerto Rico and other US territories were categorized as foreign-born. "Vigorous physical activity" was based on *Healthy People 2010* guidelines and defined as activity that causes heavy sweating or increases in breathing or heart rate for at least 20 minutes, at least 3 days per week. Body mass index (BMI) was calculated from weight and height measurements.

### Data analyses

We compared age-standardized New York City with national mean total and LDL cholesterol using the NHANES 2003-2004 fasting sample aged 20 years or older (16). We also compared the distribution of adults across various categories of total and LDL cholesterol levels.

We assigned each participant to a CHD risk group based on ATP III criteria and then estimated age-standardized prevalence of high LDL cholesterol among New York City adults overall and by ATP III risk group. To identify New York City populations at increased risk for high LDL cholesterol, we estimated age-standardized prevalence of high LDL cholesterol by demographic and health characteristics, including BMI, physical activity, smoking status, and other indicators of CHD risk.

We estimated awareness among adults with high LDL cholesterol, treatment and control among adults aware of their condition, and control among those treated with cholesterol-lowering medication. Awareness, treatment, and control of high LDL cholesterol were also examined by sociodemographic characteristics, insurance status, and ATP III risk group.

All statistical analyses were conducted by using SUDAAN version 10.0 (RTI International, Research Triangle Park, North Carolina) to account for complex survey design. Prevalence estimates were age-adjusted to the 2000 US standard population (19). Significance of univariate differences in prevalence, awareness, treatment, and control of high LDL was determined by using *t* tests derived from the general linear contrast procedure. We used χ^2^ tests to identify significant associations between the outcomes and covariates with 3 or more levels. Relative standard errors and 95% confidence intervals (CIs) were calculated for percentages. Analytic weights were poststratified to represent the New York City adult population for NYC HANES data and the US adult population for NHANES data, then further adjusted for age, sex, and race/ethnicity to address component and item nonresponse (20). Significance was set at *P* < .05.

## Results

### Mean total and LDL cholesterol

Mean total cholesterol was significantly lower in New York City (197.7 mg/dL) than in the United States overall (203.0 mg/dL) (Table 2). No difference was seen in mean LDL cholesterol overall, but New York City foreign-born adults had higher mean LDL cholesterol than US foreign-born adults (122.5 vs 117.8).

### Prevalence of ATP-defined high LDL cholesterol

Most New York City adults (64.8%; 95% CI, 61.4%-68.1%) were in the low ATP III risk group, while 17.9% (95% CI, 15.2%-20.9%) were in the high-risk group (Figure). According to ATP III-defined thresholds, total prevalence of high LDL cholesterol was 27.8% (Figure).

**Figure.**
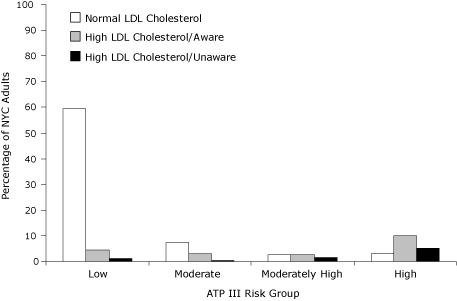
Age-standardized prevalence of high LDL cholesterol among New York City adults by ATP III risk category (5,14), New York City Health and Nutrition Examination Survey 2004. High LDL cholesterol defined as currently taking cholesterol-lowering medications or having LDL cholesterol levels at or greater than the ATP III risk-specific thresholds recommended for initiation of drug therapy: ≥100 mg/dL for the high-risk group, ≥130 mg/dL for the moderately high-risk group, ≥160 mg/dL for the moderate-risk group, and ≥190 mg/dL for the low-risk group. Abbreviations: NYC, New York City; ATP III, National Cholesterol Education Program Adult Treatment Panel III; LDL, low-density lipoprotein.

**Table:** 

**ATP III Risk Group**	**% of NYC Adults**
**Low **	
Normal LDL cholesterol	59.5
High LDL cholesterol/aware	4.3
High LDL cholesterol/unaware	1.0
**Moderate **	
Normal LDL cholesterol	7.2
High LDL cholesterol/aware	2.8
High LDL cholesterol/unaware	0.5
**Moderately high **	
Normal LDL cholesterol	2.6
High LDL cholesterol/aware	2.5
High LDL cholesterol/unaware	1.5
**High **	
Normal LDL cholesterol	2.8
High LDL cholesterol/aware	10.1
High LDL cholesterol/unaware	5.0

Significant differences in the prevalence of high LDL cholesterol were observed by ATP III risk group and across several demographic and health characteristics (Table 3). There was a strong, graded association between ATP III risk group and the presence of high LDL cholesterol: 11.1% of those at low risk, 31.3% of those at moderate risk, 56.9% of those at moderately high risk, and 78.4% of those at high risk. Older age was associated with increased risk of high LDL cholesterol; more adults aged 60 years or older (58.6%) had high LDL cholesterol than did adults aged 40 to 59 years (31.4%) and aged 20 to 39 years (6.6%). LDL cholesterol levels were higher among men than among women and among adults with high school education or less than among those with more education. A particularly low proportion of uninsured adults had high LDL cholesterol (15.4%).

More overweight (29.6%) and obese (38.9%) participants had high LDL cholesterol than did those with normal or underweight BMI (17.6%), and more current smokers had high LDL cholesterol than nonsmokers (37.2% vs 24.8%). More adults with hypertension (41.3%) or diabetes (79.1%) had high LDL cholesterol than did those without these conditions (22.4% and 22.1%, respectively).

### Awareness, treatment, and control of high LDL cholesterol

Among New York City adults with high LDL cholesterol, more than two-thirds (70.9%) were aware of their condition (Table 4). Rates of awareness varied significantly by age group, education, and health insurance status. Fewer adults aged 20 to 39 years (48.4%) and aged 40 to 59 years (65.7%) were aware of their high LDL cholesterol than those aged 60 or older (80.4%). Fewer adults with high school education or less (64.0%) were aware of their condition than those with more education (80.8%). Three-fourths (73.8%) of insured adults with high LDL cholesterol were aware of their condition, compared with 46.2% of the uninsured (*P* = .005).

Among adults who were aware of their high LDL cholesterol, most (88.1%) reported changing their diet, trying to lose weight, or increasing physical activity to lower cholesterol. A lower proportion of younger people than older people (68.0% vs 92.0%) and a higher proportion of women than men (93.4% vs 83.8%) adopted healthier behaviors. Fewer aware adults in the high-risk group were treated with medication than those in the low-risk group (62.0% vs 77.1%). Aware adults aged 60 or older (72.8%) had higher rates of treatment with medication than those aged 40 to 59 years (57.1%) and 20 to 39 years (35.7%). Only 43.7% had their LDL cholesterol adequately controlled (Table 5). We observed significant disparities in rates of control among those who were aware of their condition by risk group, age, and race/ethnicity. More than two-thirds (71.3%) of adults in the low-risk group had their LDL cholesterol controlled, compared with only one-third of adults in the high-risk group. A smaller proportion of younger adults who were aware of their high cholesterol (22.4%) had their LDL cholesterol controlled than did those aged 60 or older (51.3%). Rates of control among whites (53.2%) were higher than among blacks (31.2%) and Hispanics (31.8%).

Among those treated with cholesterol-lowering medication, two-thirds (68.3%; 95% CI, 56.9%-77.7%) had LDL cholesterol at recommended levels for their risk group. Fewer adults treated with medication in the high-risk group had their condition under control than did those in the low-risk group (53.8% vs 92.5%; *P* < .001), as were treated foreign-born adults compared with treated US-born adults (57.8% vs 78.0%, *P* = .04).

## Discussion

This study documents the large burden of high LDL cholesterol in New York City. We found that more than 1 in 4 New York City adults have high LDL cholesterol, placing them at elevated risk for CHD (2,3). This proportion is similar to national levels (8).

We also found that nearly one-third of New York City adults with high LDL cholesterol were unaware of their condition. Among those who were aware, less than two-thirds were taking cholesterol-lowering medications and less than half had their LDL cholesterol under control, suggesting that adults in New York City are not adequately treated. We found particularly low levels of treatment and control among those in the highest CHD risk groups, and low levels of control among black and Hispanic populations. Intensive efforts are needed to reduce LDL cholesterol among these groups (4).

Lower awareness and treatment rates may be due in part to providers' use of guidelines other than the 2004 ATP III. Providers who use the US Preventive Services Task Force (USPSTF) guidelines would not screen low-risk men before age 35 or women before age 45 (21). ATP III recommends screening all adults from age 20, every 5 years. Using USPSTF guidelines, 16% of those we defined as unaware of high LDL cholesterol would not have been routinely screened. Similarly, low rates of treatment in the high-risk group may be due to limited provider adoption of the 2004 ATP III guidelines, which reduced the threshold for drug therapy in the high-risk group from ≥130 mg/dL to ≥100 mg/dL. However, using the former threshold of 130 mg/dL for the high-risk group results in a similarly low treatment rate of 69.0% among those who were aware of having high LDL cholesterol.

Our analysis of the distribution of mean LDL cholesterol found similar levels between New York City and the United States, but a higher mean LDL cholesterol among foreign-born adults in New York City than among foreign-born adults in the United States overall. Foreign-born adults make up more than one-third (36.7%) of New York City's population (compared with 12.5% of the US population) (22). The higher mean in New York City may be due to differences in the racial/ethnic diversity in the NYC HANES foreign-born population (22% white, 18% black, 19% Asian, 39% Hispanic, and 2% "other") compared with the NHANES foreign-born population (27% white, 7% black, 28% Mexican American, 18% other Hispanic, and 21% "other/multiracial").

This study builds on previous NHANES studies by measuring LDL cholesterol among Hispanics and Asians, which could not be assessed in prior national surveillance data, and a closer examination of the foreign-born. New York City Hispanics had particularly low levels of awareness compared with whites, possibly because of less access to care. Lack of insurance was associated with lower awareness levels. Diagnosis of high cholesterol requires a blood test, and treatment requires ongoing monitoring, costs of which can be barriers for people without insurance coverage. Also of concern is the disparity in control rates between foreign- and US-born adults who are treated with medication. These findings support the need to identify and respond to health disparities (23) to ensure increased access to health care for all groups.

The high prevalence of high LDL coupled with poor levels of control suggests that effective strategies to reduce cholesterol requires a multilevel approach that includes and goes beyond the clinic. Population approaches to prevent and reduce high LDL cholesterol offer opportunities to reduce risk across all risk groups (24).

Our findings are consistent with those of other studies that associated overweight or obesity and smoking with high LDL cholesterol (25,26) and underscore the need for policies that improve the food environment and reduce smoking. New York City has introduced such population-based initiatives. These include restricting the use of artificial trans fat in restaurants (27), increasing the number of mobile vendors that sell vegetables, and setting nutrition standards for foods purchased by New York City agencies. Such policies aim to normalize heart-healthy eating and represent a promising strategy to reduce cholesterol levels and CHD risk (24). Smoking prevalence is dramatically lower in New York City (28) after an increase in local and state cigarette taxes (making them among the most expensive in the country), hard-hitting antitobacco advertising, and wide distribution of free nicotine patches and gum (29). Federal action to extend similar and other effective policies across the country are needed for widespread reductions in LDL cholesterol.

In addition to environment change approaches, improved clinical approaches to diagnosis and management of high LDL cholesterol are needed. Studies have demonstrated that delayed initiation of treatment for high LDL is common, despite evidence that early initiation and longer duration of therapy mitigates the atherosclerotic process (30). Once initiated, medication therapy is often not aggressive enough to reach targets because of providers' concerns about adverse effects, tolerance, and patient adherence to specific medications (31). The introduction of panel management in primary care practices is an emerging proactive systematic approach to improve care, for example, by identifying a list of the provider's patients with poorly controlled LDL for individualized outreach and follow-up by the health care team (32).

Limitations of the study include recall bias and measurement error associated with self-report. The study strictly adhered to quality assurance procedures from NHANES to limit response bias. Poststratification weighting on the basis of age group, sex, race/ethnicity, and borough was applied to decrease the effect of component and item nonresponse. Additional analyses of treatment and control of LDL cholesterol within specific subgroups may have provided a deeper understanding of disparities but were limited by sample size.

An unacceptably large proportion of the New York City population has high or uncontrolled LDL cholesterol. New York City adults who are at the greatest risk for CHD need aggressive medical intervention to reduce their LDL cholesterol levels. On a broader level, programs that address primary prevention of high cholesterol through changes in the food environment and tobacco policies can prevent CHD-related illness and death and reduce health disparities.

## Figures and Tables

**Table 1:** National Cholesterol Education Program ATP III Guidelines for Risk Groups

**ATP III Risk Group**	LDL Cholesterol Goal, mg/dL	LDL Cholesterol Level for Initiationof Therapeutic Lifestyle Changes, mg/dL	LDL Cholesterol Level for Consideration of Drug Therapy, mg/dL
High risk: CHD or CHD risk equivalents^a^ (10-year risk^b^ >20%)	<100	≥100	≥100
Moderately high risk: ≥2 risk factors^c ^(10-year risk^b^ 10%-20%)	<130	≥130	≥130
Moderate risk: ≥2 risk factors^c ^(10-year risk^b^ <10%)	<130	≥130	≥160
Low risk: 0-1 risk factor^c^	<160	≥160	≥190

Abbreviations: ATP III, Adult Treatment Panel III (5,14); LDL, low-density lipoprotein; CHD, coronary heart disease.

a Self-reported history of congestive heart failure, coronary heart disease, angina pectoris, myocardial infarction, or stroke; self-reported history of or measured diabetes.

b Framingham risk of developing angina pectoris, myocardial infarction, or CHD death (17); calculated by using ATP III algorithms.

c Self-reported cigarette smoking, hypertension, measured high-density lipoprotein (HDL) cholesterol <40 mg/dL, family history of premature CHD, and older age. Measured HDL cholesterol ≥60 mg/dL offsets the presence of 1 risk factor.

**Table 2:** Age-Standardized Mean Cholesterol Levels, NYC HANES 2004 Versus NHANES 2003-2004^a^

Characteristic	NYC HANES		NHANES		*P* Value^c^

	n^b^	Mean, mg/dL (95% CI)	n^b^	Mean, mg/dL (95% CI)	
**Total cholesterol**					
**Overall**	1,783	197.7 (195.7-199.7)	4,476	203.0 (201.5-204.4)	<.001
**Age, y**					
20-39	890	187.2 (184.4-189.9)	1,552	194.5 (192.4-196.7)	<.001
40-59	660	206.3 (203.3-209.3)	1,273	209.4 (206.0-212.8)	.15
≥60	233	202.1 (196.5-207.7)	1,651	207.0 (204.6-209.4)	.11
**Sex**					
Men	752	196.9 (194.2-199.7)	2,170	202.0 (200.4-203.6)	.002
Women	1,031	198.7 (195.9-201.5)	2,306	203.4 (200.8-206.0)	.01
**Race/ethnicity^d^**					
Non-Hispanic white	522	199.1 (195.6-202.7)	2,417	203.7 (202.1-205.2)	.02
Non-Hispanic black	385	195.2 (191.0-199.3)	871	198.5 (195.2-201.8)	.21
Asian^e^	225	200.1 (194.4-205.9)	NA	NA	NA
Hispanic^e^	622	198.3 (195.1-201.4)	NA	NA	NA
Mexican American^f^	NA	NA	900	202.4 (199.8-205)	NA
Other Hispanic^f^	NA	NA	138	207.8 (198.0-217.6)	NA
**Place of birth**					
US-born	803	194.4 (191.6-197.2)	3,546	202.5 (200.9-204.1)	<.001
Foreign-born (includes US territories)	974	200.8 (198.1-203.6)	929	205.3 (200.9-209.8)	.08
**LDL cholesterol **					
**Overall**	1,286	119.8 (117.7-121.9)	1,900	118.1 (115.7-120.5)	.28
**Age, y**					
20-39	643	111.0 (108.1-113.8)	652	113.5 (110.1-116.9)	.24
40-59	483	127.5 (123.7-131.2)	533	122.1 (117.1-127.0)	.07
≥60	160	122.6 (116.6-128.5)	715	119.6 (116.3-123.0)	.39
**Sex**					
Men	538	122.4 (119.3-125.4)	905	118.9 (115.6-122.2)	.12
Women	748	118.1 (115.3-120.8)	995	117.1 (114.1-120.2)	.64
**Race/ethnicity^d^**					
Non-Hispanic white	372	118.9 (115.2-122.7)	1,042	118.6 (116.0-121.2)	.88
Non-Hispanic black	281	118.5 (113.8-123.2)	360	116.0 (111.8-120.2)	.42
Asian^e^	158	121.2 (113.6-128.8)	NA	NA	NA
Hispanic^e^	454	121.9 (118.2-125.5)	NA	NA	NA
Mexican American^f^	NA	NA	383	120.1 (116.9-123.4)	NA
Other Hispanic^f^	NA	NA	51	117.3 (105.0-129.6)	NA
**Place of birth**					
US-born	575	116.8 (113.6-120.1)	1,501	118.1 (115.3-120.9)	.55
Foreign-born (includes US territories)	705	122.5 (119.4-125.6)	399	117.8 (114.2-121.5)	.046

Abbreviations: NYC HANES, New York City Health and Nutrition Examination Survey; NHANES, National Health and Nutrition Examination Survey; CI, confidence interval; NA, not applicable; LDL, low-density lipoprotein.

a All estimates age-adjusted to the 2000 US standard population.

b Totals may differ because of nonresponse to specific questions.

c Pairwise comparisons of New York City to US population based on general linear contrast procedure.

d "Other race" not presented because of small numbers.

e NHANES does not provide estimates for Hispanics or Asians.

f NYC HANES does not provide estimates for Mexican Americans or "other Hispanics."

**Table 3:** Age-Standardized Prevalence of ATP III-Defined High LDL Cholesterol, NYC HANES 2004^a^

**Characteristic**	n^b^	% (95% CI)	*P* Value^c^
**Total**	1,271	27.8 (25.1-30.8)	NA
**ATP III risk group**			<.001
High risk	174	78.4 (69.3-85.4)	<.001
Moderately high risk	65	56.9 (32.6-78.3)	.001
Moderate risk	135	31.3 (22.8-41.3)	<.001
Low risk	897	11.1 (7.9-15.3)	Reference
**Age, y**			<.001
20-39	636	6.6 (4.7-9.1)	<.001
40-59	477	31.4 (26.6-36.6)	<.001
≥60	158	58.6 (50.2-66.5)	Reference
**Sex**			
Men	533	34.5 (30.4-38.8)	Reference
Women	738	22.4 (19.2-25.9)	<.001
**Race/ethnicity^d^**			.08
Non-Hispanic white	370	28.1 (23.8-32.9)	Reference
Non-Hispanic black	276	28.7 (22.9-35.2)	.89
Non-Hispanic Asian	157	29.6 (21.1-39.8)	.78
Hispanic	451	25.3 (21.1-30.1)	.38
**Place of birth**			
US-born	568	27.5 (23.6-31.9)	Reference
Foreign-born (includes US territories)	702	27.7 (23.9-31.9)	.95
**Annual household income, $**			
<20,000	424	29.2 (24.5-34.4)	.47
≥20,000	824	26.9 (23.5-30.6)	Reference
**Education**			
High school or less	620	32.9 (29.1-36.9)	.001
More than high school	649	23.4 (19.6-27.7)	Reference
**Health insurance status**			
Insured	937	29.8 (26.8-33.0)	Reference
Uninsured	331	15.4 (10.0-23.0)	<.001
**Body mass index, kg/m^2^**			<.001
Normal/underweight (<25.0)	509	17.6 (13.7-22.2)	Reference
Overweight (25.0-29.9)	436	29.6 (25.1-34.5)	<.001
Obese (≥30.0)	324	38.9 (34.1-44.0)	<.001
**Vigorous physical activity**			
≥20 min, 3 d/week	322	26.8 (20.8-33.9)	.68
<20 min, 3 d/week	946	28.4 (25.2-31.7)	Reference
**Smoking**			
Current smoker (last 30 days)	313	37.2 (31.7-43.2)	<.001
Nonsmoker	957	24.8 (21.8-28.1)	Reference
**Diabetes**			
Has diabetes	120	79.1 (67.9-87.2)	<.001
No diabetes	1,150	22.1 (19.3-25.2)	Reference
**Hypertension**			
Has hypertension	227	41.3 (34.1-48.9)	<.001
No hypertension	1,042	22.4 (18.9-26.3)	Reference

Abbreviations: ATP III, Adult Treatment Panel III (5,14); LDL, low-density lipoprotein; NYC HANES, New York City Health and Nutrition Examination Survey; CI, confidence interval; NA, not applicable.

a High LDL cholesterol defined as currently taking cholesterol-lowering medications or having LDL cholesterol levels at or greater than the ATP III risk-specific cutpoints recommended for initiation of drug therapy. All estimates age-adjusted to the 2000 US standard population.

b Totals may differ because of nonresponse to specific questions.

c χ^2^ test of independence computed for variables with 3 or more levels. Pairwise comparisons to reference group based on general linear contrast procedure.

d "Other race" not presented because of small numbers.

**Table 4:** Awareness of High LDL Cholesterol, NYC HANES 2004

**Characteristic**	n^a^	% (95% CI)	*P* Value^b^
**Total**	269	70.9 (64.2-76.7)	NA
**ATP III risk group**			.06
High risk	141	66.8 (57.7-74.8)	.09
Moderately high risk	40	63.1^c^ (46.4-77.2)	.10
Moderate risk	37	83.7^c^ (67.1-92.9)	.64
Low risk	51	79.8 (64.5-89.5)	Reference
**Age, y**			.009
20-39	36	48.4^c^ (30.3-67.1)	.003
40-59	146	65.7 (56.6-73.8)	.02
≥60	87	80.4 (69.7-88.0)	Reference
**Sex**			
Men	145	67.6 (58.5-75.5)	Reference
Women	124	75.5 (66.5-82.7)	.15
**Race/ethnicity^d^**			.30
Non-Hispanic white	91	78.1 (66.1-86.8)	Reference
Non-Hispanic black	62	67.2 (54.4-77.8)	.17
Non-Hispanic Asian	33	65.2^c^ (48.7-78.8)	.14
Hispanic	82	63.2 (51.3-73.7)	.08
**Place of birth**			
US-born	113	73.7 (63.4-81.9)	Reference
Foreign-born (includes US territories)	155	68.3 (59.7-75.8)	.38
**Education**			
High school or less	166	64.0 (54.9-72.1)	.006
More than high school	102	80.8 (71.8-87.5)	Reference
**Annual household income, $**			
<20,000	100	65.6 (54.5-75.2)	.26
≥20,000	161	72.8 (65.1-79.5)	Reference

Abbreviations: LDL, low-density lipoprotein; NYC HANES, New York City Health and Nutrition Examination Survey; CI, confidence interval; NA, not applicable; ATP III, Adult Treatment Panel III (5,14).

a Totals may differ because of nonresponse to specific questions.

b χ^2^ test of independence computed for variables with 3 or more levels. Pairwise comparisons to reference group based on general linear contrast procedure.

c Estimate should be interpreted with caution. Estimate's relative standard error (a measure of estimate precision) is >30% or sample size is <50, making the estimate potentially unreliable.

d "Other race" (n = 17) not presented because of small numbers.

**Table 5:** Treatment and Control of High LDL Cholesterol Among Respondents Aware of Their Status, NYC HANES 2004

**Characteristic**	n^a^	Adopted Healthier Behavior % (95% CI)	*P* Value^b^	Treated With Medication % (95% CI)	*P* Value^b^	**Controlled** c % (95% CI)	*P* Value^b^
**Total**	180	88.1 (82.3-92.2)	NA	64.0 (55.4-71.8)	NA	43.7 (34.4-53.5)	NA
**ATP III risk group**			.08	—	.17	—	.03
High risk	88	93.9 (86.7-97.3)	.17	62.0 (49.5-73.1)	.12	33.3 (22.0-47.0)	.001
Moderately high risk	21	83.5^d^ (60.6-94.4)	.86	46.2^d^ (23.4-70.8)	.04	38.9^d^ (17.4-65.7)	.04
Moderate risk	31	75.8^d^ (55.8-88.6)	.35	66.7^d^ (45.3-82.9)	.39	42.1^d^ (24.7-61.7)	.03
Low risk	40	85.4^d^ (70.7-93.4)	Reference	77.1^d^ (60.2-88.3)	Reference	71.3^d^ (51.0-85.6)	Reference
**Age, y**			.16	—	.05	—	.07
20-39	18	68.0^d^ (41.2-86.6)	.06	35.7^d^ (16.0-61.8)	.009	22.4^d^ (8.2-48.4)	.02
40-59	94	86.4 (76.7-92.4)	.26	57.1 (45.0-68.3)	.08	37.2 (27.3-48.2)	.11
≥60	68	92.0 (83.3-96.4)	Reference	72.8 (58.9-83.4)	Reference	51.3 (37.3-65.1)	Reference
**Sex**							
Men	89	83.8 (74.2-90.3)	Reference	64.3 (52.8-74.4)	Reference	46.7 (34.2-59.6)	Reference
Women	91	93.4 (85.9-97.0)	.049	63.6 (51.6-74.1)	.93	39.9 (29.4-51.6)	.39
**Race/ethnicity^e^**			.28	—	.11	—	.14
Non-Hispanic white	70	89.3 (80.4-94.4)	Reference	67.2 (53.8-78.2)	Reference	53.2 (39.9-66.0)	Reference
Non-Hispanic black	40	85.7^d^ (69.3-94.1)	.62	56.1^d^ (38.2-72.5)	.32	31.2^d^ (16.4-51.1)	.047
Non-Hispanic Asian	20	96.4^d^ (77.1-99.5)	.13	83.6^d^ (63.4-93.7)	.08	47.5^d^ (25.7-70.3)	.65
Hispanic	50	83.7 (69.0-92.2)	.42	55.5 (41.5-68.7)	.22	31.8 (19.2-47.7)	.03
**Place of birth**							
US-born	80	88.0 (79.4-93.3)	Reference	64.5 (50.5-76.4)	Reference	50.3 (36.5-64.1)	Reference
Foreign-born (includes US territories)	100	88.2 (79.2-93.6)	.97	63.5 (53.0-72.8)	.91	36.7 (26.6-48.1)	.12
**Education**							
High school or less	102	84.3 (75.0-90.6)	.11	63.0 (51.3-73.3)	.77	39.7 (28.9-51.6)	.27
More than high school	78	92.3 (84.1-96.4)	Reference	65.1 (53.4-75.3)	Reference	48.1 (35.7-60.8)	Reference
**Annual household income, $**							
<20,000	62	93.4 (85.0-97.2)	.05	67.3 (52.5-79.3)	.37	45.3 (32.0-59.2)	.54
≥20,000	112	84.4 (76.1-90.2)	Reference	59.3 (48.1-69.7)	Reference	39.7 (28.4-52.3)	Reference

Abbreviations: LDL, low-density lipoprotein; NYC HANES, New York City Health and Nutrition Examination Survey; CI, confidence interval; NA, not applicable; ATP III, Adult Treatment Panel III (5,14).

a Totals may differ because of nonresponse to specific questions.

b χ^2^ test of independence computed for variables with 3 or more levels. Pairwise comparisons to reference group based on general linear contrast procedure.

c "Controlled" is defined as <100 mg/dL for the high risk group, <130 mg/dL for the moderately high risk group, <130 mg/dL for the moderate risk group, and <160 mg/dL for the low risk group.

d Estimate should be interpreted with caution. Estimate's relative standard error (a measure of estimate precision) is >30% or sample size is <50, making the estimate potentially unreliable.

e "Other race" (n = 17) not presented because of small numbers.
